# Parallel Grooved Microstructure Manufacturing on the Surface of Si_3_N_4_ Ceramics by Femtosecond Laser

**DOI:** 10.3390/mi15030394

**Published:** 2024-03-14

**Authors:** Xufeng Wen, Yanfeng Gao, Hua Zhang, Yaxin Yang

**Affiliations:** School of Mechanical and Automotive Engineering, Shanghai University of Engineering Science, Shanghai 201620, China; w_xfwork@163.com (X.W.); hzhang@sues.edu.cn (H.Z.); 18835902001@163.com (Y.Y.)

**Keywords:** femtosecond laser, grooved microstructure, Si_3_N_4_ ceramics, machining

## Abstract

Machining special microstructures on the surface of silicon nitride ceramics helps improve their service performance. However, the high brittleness and low fracture toughness of silicon nitride ceramics make it extremely difficult to machine microstructures on their surface. In this study, a femtosecond laser is used to machine parallel grooved microstructures on the surface of silicon nitride ceramics. The effects of the laser polarization angle, laser single pulse energy, scanning line spacing, and laser scan numbers on the surface morphology and geometric characteristics of grooved microstructures are researched. It is found that a greater angle between the direction of the scanning path and laser polarization is helpful to obtain a smoother surface. As the single pulse energy increases, debris and irregular surface structures will emerge. Increasing the laser scan line spacing leads to clearer and more defined parallel grooved microstructures. The groove depth increases with the increase in the scan numbers. However, when a certain number of scans is reached, the depth will not increase further. This study serves as a valuable research foundation for the femtosecond laser processing of silicon nitride ceramic materials.

## 1. Introduction

Silicon nitride (Si_3_N_4_) ceramic is one of the important materials in the fields of aerospace and military industry because of its high temperature stability, excellent wear and corrosion resistance, good insulation and electromagnetic wave transparency [1,2,3,4]. For improving the performance of aerospace or missile products, such as hydrophobicity and self-cleaning properties, some special microstructures need to be manufactured on the surface of silicon nitride materials [5,6]. However, the high brittleness and low fracture toughness make it extraordinarily difficult to machine microstructures on the surface of silicon nitride ceramics [7].

In recent years, the femtosecond laser has been widely studied for machining silicon nitride materials [8]. As a non-contact processing technology, a femtosecond laser adopting an ultra-short pulse laser ablates the materials from the surface of products. In the process of laser ablation [9,10], laser energy is absorbed by free electrons, causing lattice heating and atomic ionization, and the probability of brittleness and even pulverization is significantly reduced [11,12]. Therefore, a femtosecond laser is an ideal means of machining microstructures on the surface of silicon nitrides [13].

Konig W et al. [14] applied laser-assisted machining technology for ceramic processing and made a preliminary study on the CO_2_ laser cutting of silicon nitride ceramics. They found that when the workpiece surface temperature reached more than 1100 °C the roughness was less than one micron and there were no cracks. Chen et al. [9] studied the grooved structure on the surface of silicon nitride using a pulsed laser with different specifications and explained the effect of single parameters of a pulsed laser on the surface of silicon nitride ceramics. The results showed that a change in surface laser power had a significant impact on the surface of silicon nitride ceramics. With the increase in laser power, the depth of the effect on the material increased. When the laser power was increased to 35 W, the maximum increase in laser depth was 181.3%. Wu et al. [15] studied the laser heating-assisted cutting of silicon nitride ceramics technology, utilizing a laser to improve the temperature of the local material, change the processing performance of the material, reduce tool wear, increase processing quality, and improve processing efficiency. Liu et al. [16] studied the effect of the laser machining surface micro-texture on the friction and wear performance of ceramic cutting tools and concluded that the machining micro-texture can reduce the friction coefficient of the tool surface, improve the wear resistance, and make the stress distribution more uniform. Wang et al. [17] studied the effect of nano-laser drilling on silicon nitride ceramics with different scanning intervals and found that the surface roughness of the hole cross-section increases with the increase in scanning interval. Yao et al. [18] studied a solution-assisted laser processing method. The study showed that water-jet-assisted laser processing effectively reduced the energy of the laser ablation of the material. When the laser current was 200 A, the frequency was 50 Hz, and the pulse width was 0.6 ms, the groove depth was reduced by 30% and the groove width was increased by 21%. Yu et al. [19] studied the femtosecond laser ablation of silicon nitride in different environments and analyzed the effects of water environments on surface size and morphology. They concluded that when using a low-temperature assisted laser for multi-layer laser ablation, water-constrained laser processing cannot produce significant ablation depths. Wu et al. [20] studied the grinding mechanism of laser-assisted grinding (LAG) on silicon nitride ceramics, focusing on the preparation of micro grooves and morphology analysis. The results showed that the LAG process can significantly improve grinding quality and reduce grinding force. Zheng et al. [21] used a laser-assisted grinding method to machine checkerboard, circular, and circular arc micro-textures on the cutting tool of silicon nitride materials. The experimental results show that this method can improve the machining quality of silicon nitride ceramic tools by 6%. Kai Liao et al. [22] used femtosecond laser direct writing technology to prepare a drag-reducing superhydrophobic functional structure with a micro-column array at the bottom of a quartz glass microchannel. After multiple experiments, it was found that this micro-texture could significantly reduce flow resistance. Zhai et al. [23] studied the effect of femtosecond laser processing on the bonding strength of stainless-steel coatings with micro textures. The experiment showed that processing numbers have the greatest impact on micro grooves, followed by scanning speed and laser power. The processed surface increased the bonding force between the coating and the substrate, extending the lifespan of the substrate. These studies provide a theoretical basis for the femtosecond laser processing of silicon nitride.

Currently, numerous research studies are investigating the application of femtosecond laser technology to enhance the cutting capabilities and prolong the lifespan of metal tools. In addition, some scholars are committed to studying the different results of processing silicon nitride in different environments. However, there is a noticeable scarcity of research focusing on the various parameters involved in femtosecond laser processing of silicon nitride materials. To delve deeper into the realm of femtosecond laser processing of silicon nitride materials, this research article investigates the impact of various parameters, including the femtosecond laser single pulse energy, scanning line spacing, scanning path direction, and the laser scan numbers on the surface morphology of silicon nitride ceramics. Through a meticulous analysis of the surface morphology and geometric attributes of the processed silicon nitride ceramics, the effects of the single parameters of a pulsed laser on the processed silicon nitride ceramics were described.

## 2. Experiment

### 2.1. The Experimental System

**Figure 1:**
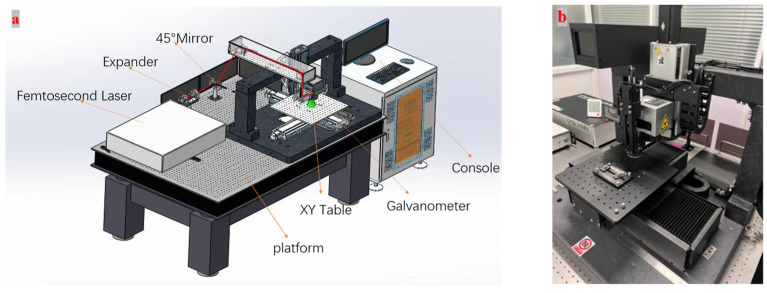
FemtoYL-40 Infrared femtosecond laser. (**a**) Integral composition. (**b**) Processing platform.

Figure 1 shows the experiment system of this study. A FemtoYL-40 Infrared femtosecond laser power is used in this experiment. The femtosecond laser beam machining system consists of an infrared ultra-fast laser, a beam expander, a scanning galvanometer, and a field lens. The femtosecond laser was produced by Wuhan Core Tech Intelligent Equipment Technology Co., Ltd. (Wuhan, China). The beam is focused on the surface of a silicon nitride ceramic through a 100 mm × 100 mm field lens. The technical parameters of the femtosecond laser power are shown in Table 1. The silicon nitride plate is mounted on a precision motion platform. The platform adopts a high-precision linear motor and the stroke is 200 mm × 200 mm. The technical parameters of the femtosecond laser power are shown in Table 1. A Hitachi S-3400N Scanning Electron Microscope (SEM) (Hitachi Ltd., Tokyo, Japan) is used to observe the micro-morphology of the machined surface. An Olympus Ols500 (Olympus Corporation, Tokyo, Japan) confocal microscope is used to measure the geometrical morphology of the grooved microstructure. The measured data were averaged through five repeated experiments.

### 2.2. The Experimental Materials

**Figure 2:**
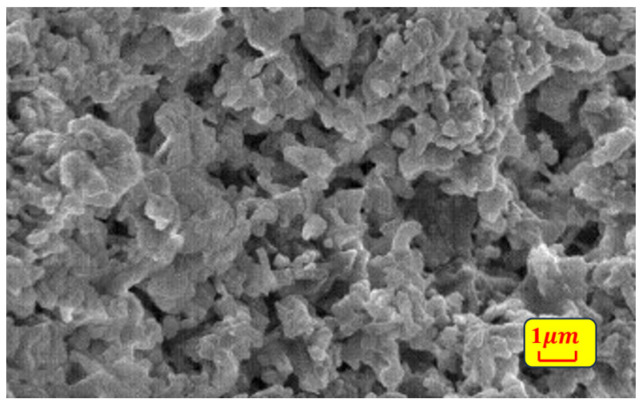
SEM morphology of Si_3_N_4_ plate before laser processing.

In the experiments, 15 mm × 15 mm × 2 mm silicon nitride plates were used for processing, which were purchased from Fuzhou Kunpeng Optoelectronic Technology Co., Ltd. (Fuzhou, China). The physical properties of the used silicon nitride are shown in Table 2 and the original micro-morphology of it is shown in Figure 2.

### 2.3. The Experimental Parameters

**Figure 3:**
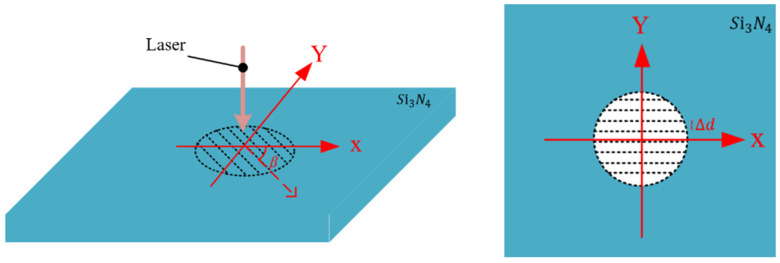
The laser scan path and the laser polarization direction.

Figure 3 shows the laser scan path, the laser polarization direction (perpendicular to the Y-axis), and the included angle β between them. The experimental parameters adopted in this study are shown in Table 3.

## 3. Experimental Results and Discussion

### 3.1. The Influence of Laser Polarization Angle

#### Experimental Results

**Figure 4:**
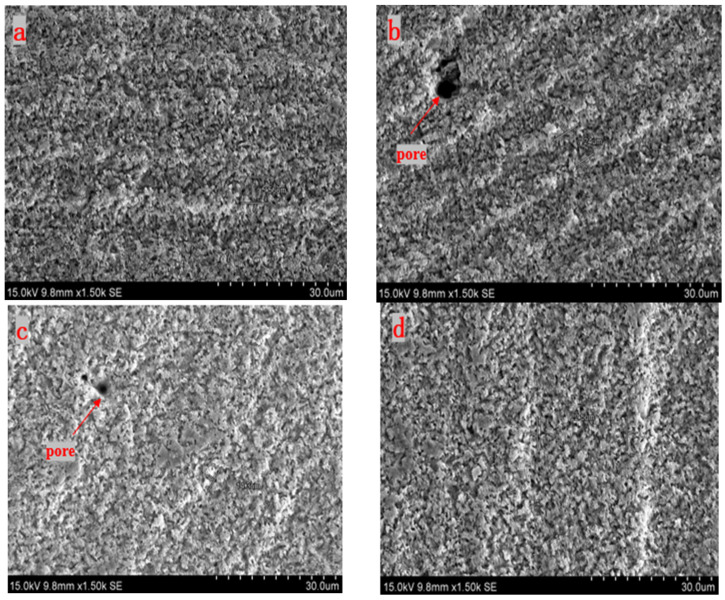
SEM images of treated surface morphologies under different scanning paths (**a**) β = 0°, (**b**) β = 30°, (**c**) β = 60°, and (**d**) β = 90°.

In the experiments, the angles *β*, between the direction of the laser scanning path and the laser polarization, are set to 0°, 30°, 60°, and 90°. The femtosecond laser single energy is set to 10 μJ, the scan line spacing is set to 5 μm, and the laser scan number is set to one time. The experimental results are shown in Figure 4.

It is observed that, compared with Figure 4c,d, the parallel grooved microstructures in Figure 4a,b are relatively clear. Although, in Figure 4b,c, some pores are formed on the surface of the silicon nitride. Generally, with the increase in *β*, the grooves on the surface of the silicon nitride gradually disappear, indicating that the greater the angle between the direction of the scanning path and the direction of polarization, the smoother the obtained machined surface will be. The results show that the angle between the scanning path and the laser polarization direction has a significant influence on the surface of the silicon nitride.

**Figure 5:**
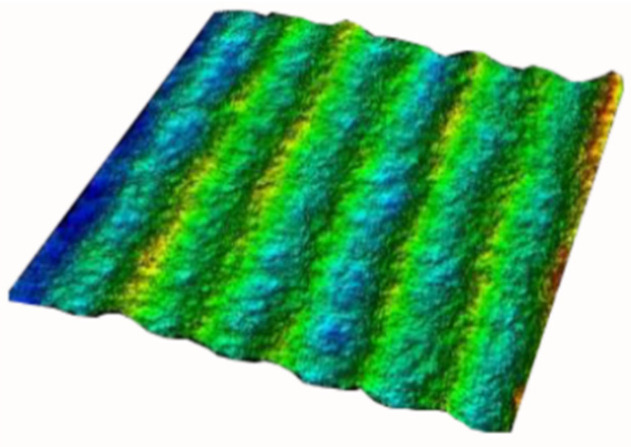
Geometric image of processed surface.

**Figure 6:**
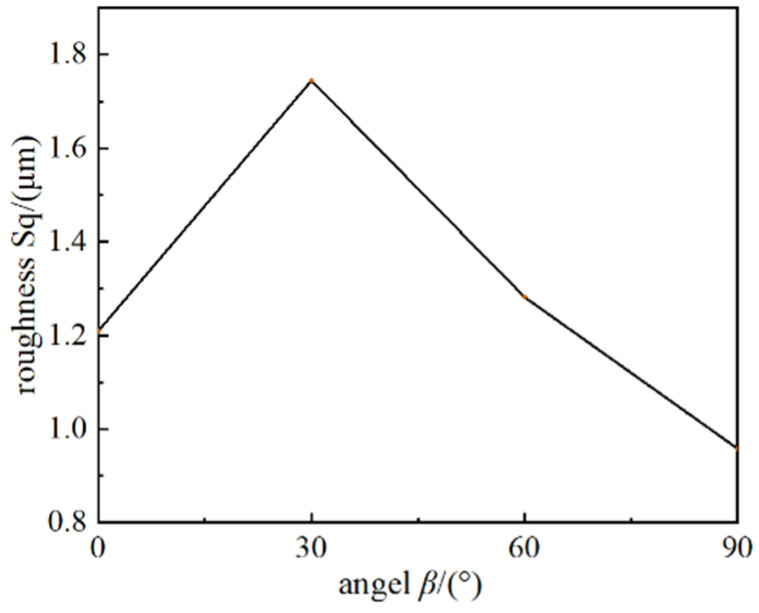
Roughness of the surface under different laser polarization angles.

To further study the influence of the laser polarization angle, the micro-geometrical shapes of the surfaces are measured using the confocal microscope method. The three-dimensional geometrical images are shown in Figure 5, and the roughness of the surface processed under different laser polarization angles is shown in Figure 6.

Figure 6 shows that the surface roughness reaches the minimum value of 0.985 μm when the angle *β* is 90°, and the surface roughness reaches the maximum value of 1.744 μm when *β* is 30°. It further confirms that a larger angle between the scan path direction and the polarization can make the machined surface smoother.

### 3.2. Influence of Laser Single Pulse Energy

#### 3.2.1. Experimental Results

**Figure 7:**
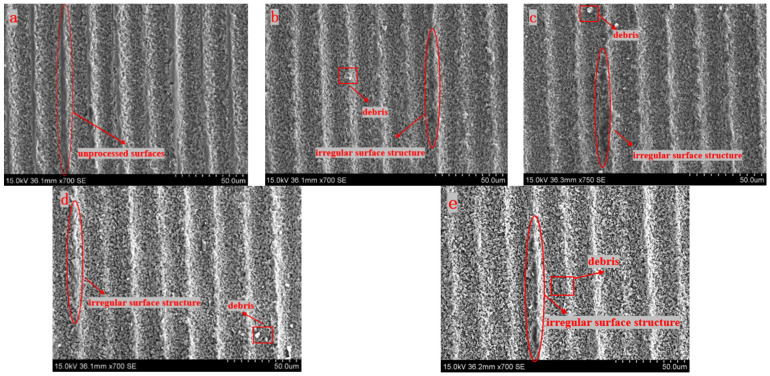
SEM images of the surface under different single energy processing (**a**) 5 μJ, (**b**) 10 μJ, (**c**) 15 μJ, (**d**) 20 μJ, and (**e**) 30 μJ.

In the experiments, the femtosecond laser single energies are set to 5 μJ, 10 μJ, 15 μJ, 20 μJ, and 30 μJ. The angle *β* between the direction of the laser scanning path and the laser polarization is set to 90°, the scan line spacing is set to 20 μm, and the laser scan number is set to one. The experimental results are shown in Figure 7.

It can be clearly seen that all the surfaces processed with different single energies have obvious parallel grooves. Compared with the other images, Figure 7a has a lot of unprocessed surfaces. This is because the central energy of the laser at this time is enough to ablate the silicon nitride material, while the edge energy is too small to reach the ablation threshold of silicon nitride. So that a large number of unprocessed surfaces are retained. Compared with Figure 7a, some irregular surface structures and debris exist in the other images. This is primarily due to the fact that the plasma wave caused by an energy density that is too high may produce a reverse impact force, which will lead to unstable laser processing, and make the edge of the surface micro-groove uneven.

**Figure 8:**
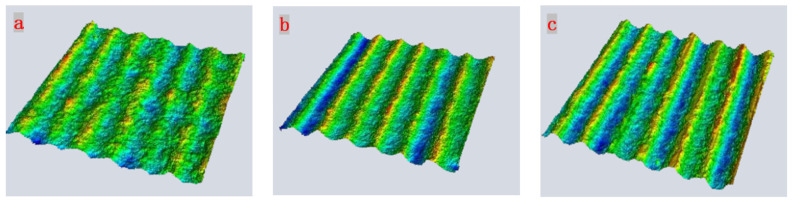
Three-dimensional geometric images (**a**) 5 μJ, (**b**) 20 μJ, and (**c**) 30 μJ.

**Figure 9:**
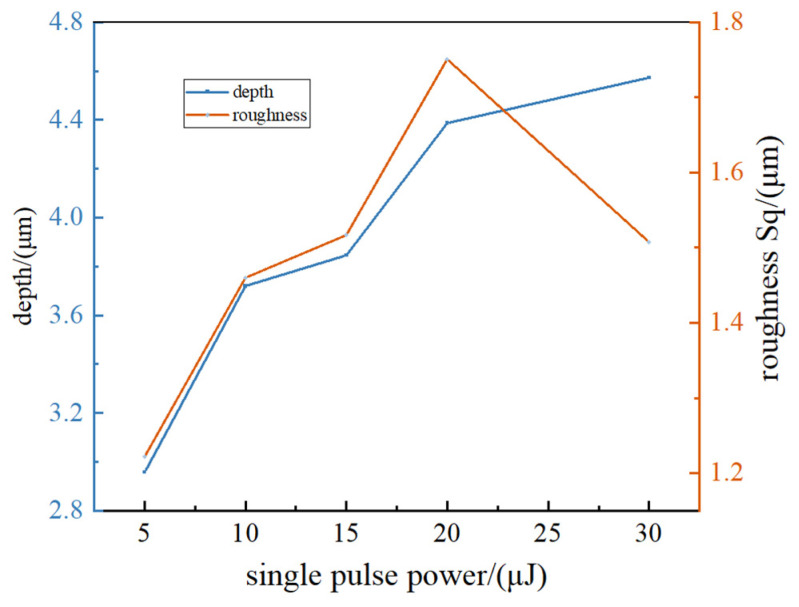
The groove depth and surface roughness under different laser single energies.

To further study the influence of the laser single pulse energy, the micro-geometrical shapes of the surfaces are measured with the confocal microscope method. The three-dimensional geometrical images are shown in Figure 8, and the roughness and depth of the surfaces processed under different single pulse energies are shown in Figure 9.

Since the femtosecond laser beam is a Gaussian beam, the center energy density is the largest, and it can be clearly seen from Figure 8 that the microgroove is in a parabolic shape. The grooves in each sample are not the same, the depth of all grooves is measured, and the maximum and minimum values are removed to obtain an average value. Figure 9 shows that, with the increase in single energy, the depth of the microgroove increases. It can also be seen that the surface roughness reaches a maximum value when the single energy is 20 μJ.

#### 3.2.2. Discussion

**Figure 10:**
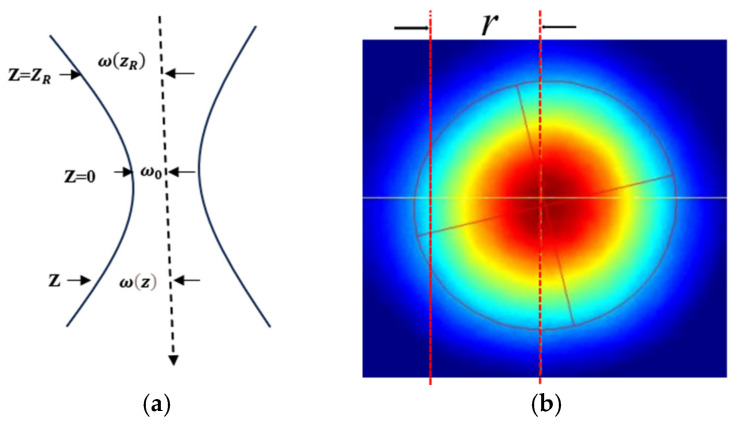
Schematic illustration of the profile and light intensity distribution of a Gaussian beam. (**a**) Contour of Gaussian beam. (**b**) Light intensity distribution on the cross-section of a Gaussian beam.

Femtosecond fiber laser beams are generally approximated as Gaussian light beams, and the light intensity distribution of the beam cross-section is a Gaussian function, as shown in Figure 10. The light intensity can be expressed by Equation (1) [24]

(1) $$l\left(r\right)=I_{0}e^{\frac{-2r^{2}}{{{(\omega }_{z})}^{2}}}$$

In Equation (1), *r* is the distance from the optical axis within the cross-section of the beam; $\omega_{z}$ is the radius when the amplitude of the electric field is 1/*e* on the axis, called the spot size; 2$\omega_{z}$ is the diameter of the spot. When a Gaussian beam propagates, the intensity distribution of each cross-section is still a Gaussian function, but the width of the intensity profile along the optical axis changes to the minimum width at the waist, with a diameter of 2$\omega_{0}$. The variation pattern of the spot size along the optical axis is:

(2) $$\left\{\begin{array}{c} \omega \left(z\right)=\omega_{0}\sqrt{1+{\left(\frac{z}{Z_{R}}\right)}^{2}}\\ z_{R}=\frac{\pi \omega_{0}^{2}}{\lambda } \end{array}\right.$$

In the equation, $z_{R}$ is the Rayleigh length; λ is the laser wavelength. At the Rayleigh length, the spot area is twice the waist area, $\omega (z_{R})$ = $\sqrt{2}\omega_{0}$ [25]. Additionally, as the cross-section moves away from the waist position, the laser energy will gradually diverge and decrease [26].

If the power in a barrel within radius *r* is *P*(*r*), then:

(3) $$P(r)=\int_{0}^{r}2\pi tI\left(t\right)dt=\frac{\pi }{2}\omega {\left(z\right)}^{2}I_{0}\left(z\right)\left\{1-\exp(-2\frac{r^{2}}{\omega {\left(z\right)}^{2}})\right\}$$

Then the total power is:

(4) $$P(r)=\underset{r\to \infty }{\lim}\frac{\pi }{2}\omega {\left(z\right)}^{2}I_{0}\left(z\right)\left\{1-\exp(-2\frac{r^{2}}{\omega {\left(z\right)}^{2}})\right\}=\frac{\pi }{2}\omega {\left(z\right)}^{2}I_{0}\left(z\right)$$

The total power of the Gaussian beam, *P*(*r*), is equal to half the product of the area at the waist of the beam and the maximum light intensity, so:

(5) $$I_{0}\left(z\right)=\frac{2P(r)}{\pi \omega {\left(z\right)}^{2}}$$

Therefore [21], the general distribution of light intensity on the propagation path of a femtosecond laser is:

(6) $$I_{0}\left(z,r\right)=\frac{2P\left(r\right)}{\pi \omega {\left(z\right)}^{2}}\exp(-\frac{2r^{2}}{{{(\omega }_{z})}^{2}})$$

Figure 10b shows the light intensity distribution of the cross-section of a Gaussian beam. The red energy in the center is the highest, and the blue energy at the edge is the lowest. During processing, the ablation depth of the middle part is deep, and the ablation depth of the edge area is shallow, which may not be ablated. Therefore, we can see that Figure 7a has a lot of unprocessed surfaces and the microgroove is in a parabolic shape.

### 3.3. The Influence of Laser Scan Line Spacing

#### 3.3.1. Experimental Results

**Figure 11:**
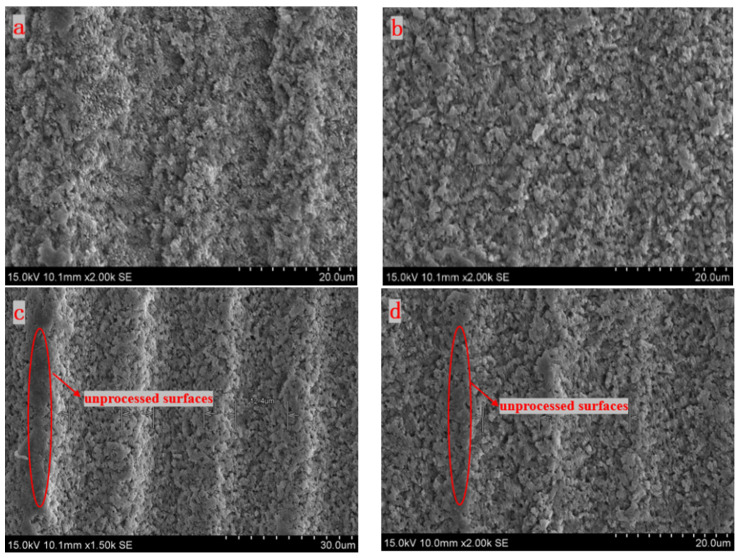
SEM image of the surface processed under different scanning line spaces (**a**) 2 μm, (**b**) 5 μm, (**c**) 15 μm, and (**d**) 20 μm.

In the experiments, the scan line spacings are set at 2 μm, 5 μm,15 μm, and 20 μm. The femtosecond laser single energy is set to 10 μJ, the direction of the laser scanning path and the laser polarization is set to 90°, and the laser scan number is set to one. The experimental results are shown in Figure 11.

It is observed that, compared with Figure 11a,b, the parallel grooved microstructures in Figure 11c,d are relatively clear. With the increase in laser scan line space, the parallel grooved microstructures become clearer and clearer. The experimental explanation shows that reducing the spacing between the scanning lines can smooth the machined surface of the sample. This is closely related to the overlap of spot energy within the same cross-section.

**Figure 12:**
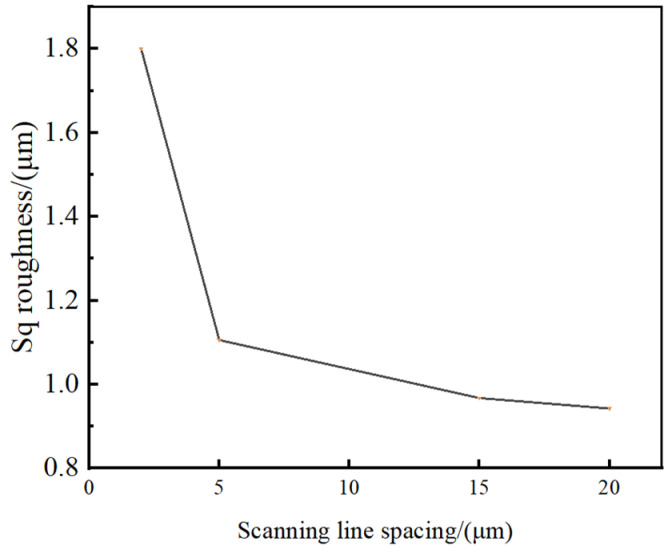
Roughness (Sq) versus scanning line interval.

To further study the influence of the laser scan line spacing, the surface roughness is measured using the confocal microscope method. The Sq roughness versus scanning line interval is shown in Figure 12.

Figure 12 shows that the surface roughness reaches the minimum value of 0.943 μm when the scanning line spacing is 20 μm, and the surface roughness reaches the maximum value of 1.800 μm when the scanning line spacing is 2 μm. The surface roughness decreases exponentially with the increase in scan line spacing.

#### 3.3.2. Discussion

When the scanning line spacing is 2 μm, the overlap rate of the spot is 75–80%. When the scanning line spacing is 5 μm, the overlap rate of the spot is 60–65%. When the scanning line spacing is greater than or equal to 15 μm, the spot overlap rate is 0.

Due to the scanning line spacing being smaller than the spot diameter, there is a partial overlap between the scanning paths within the same cross-section, resulting in the overlap of spot energy at different times. The expression for the distribution of superimposed light intensity on the machined surface of silicon nitride within the same cross-sectional area is given in Equation (7) [24]:

(7) $$I\left(z,r+∆d\right)=\frac{2P\left(r\right)}{\pi \omega {\left(z\right)}^{2}}\exp(-\frac{2{\left(r-∆d\right)}^{2}}{{{(\omega }_{z})}^{2}})$$

Therefore, the lower the overlap rate, the clearer the parallel grooved microstructures become, and, in the same cross-section, the superposition intensity of the femtosecond laser decreases exponentially with the increase in the scan line spacing, so, the surface roughness decreases exponentially with the increase in scan line spacing.

## 4. The Influence of the Scan Numbers of Laser

### Experimental Results

**Figure 13:**
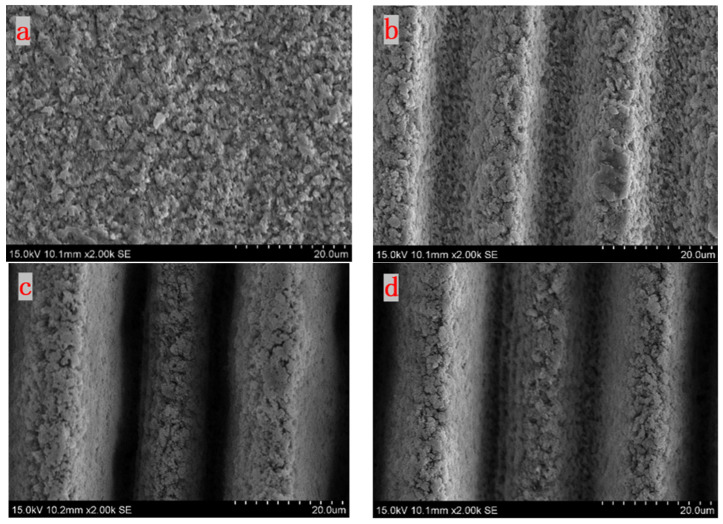
SEM image of the surface processed under different scan numbers (**a**) 1 time, (**b**) 5 times, (**c**) 15 times, and (**d**) 25 times.

In the experiments, the scan numbers of the laser are set to 1, 5, 15, and 20. The angles *β* between the direction of the laser scanning path and the laser polarization are set to 90°, the scan line spacing is set to 5 μm, and the femtosecond laser single energy is set to 10 μJ. The experimental results are shown in Figure 13.

It is observed that there is no clear groove when the number of scans is one, that an obvious groove appears from the fifth time, and that the outline of the groove becomes clearer and clearer. To further investigate the influence of scan numbers on geometric characteristics, the groove depths are measured. It is found that the groove depth reaches 15.959 μm when employing 15 scan times. Subsequently, with 25 scan times, the groove depth increases slightly to 16.179 μm. This trend can be attributed to the principles outlined in Equation (2), which describe how laser energy gradually diverges and diminishes as the cross-section moves away from the waist.

## 5. Conclusions

In this paper, the influence of femtosecond laser scan path directions, single pulse energies, scan spacings, and scan numbers on the surface morphology and geometric characteristics of silicon nitride ceramics are researched. The following conclusions are obtained.

1. When the scanning path direction is perpendicular to the polarization direction, the surface roughness of silicon nitride is reduced. With an increase in the angle between the scanning path direction and the polarization direction, the width of the groove gradually increases and the grooves on the surface of silicon nitride become unclear and gradually disappear.
2. When the single pulse energy is small there are many untreated surfaces. With the increase in the single pulse energy, debris and irregular surface structure will appear.
3. With the increase in the laser scan line spacing, the parallel grooved microstructures become clearer and clearer. The surface roughness reaches the minimum value of 0.943 μm when the scanning line spacing is 20 μm, and the surface roughness reaches the maximum value of 1.800 μm when the scanning line spacing is 2 μm.
4. There is no clear groove when the scan number is one, and an obvious groove appears when the scan number reaches five. The depth of the groove linearly increases with the scan number when the scan number is less than 15. Whereas, when the scan number is larger than 15, the groove depth increases very slowly.

## Figures and Tables

**Table 1:** The technical parameters of the femtosecond laser power.

Parameters	Values
Center wavelength	1035 ± 5 nm
Pulse duration	0.8 ps
Frequency	200 kHz
Maximum power	50 W
Spot diameter in focal plane	13 μm
Number of pluses per dot	1
Scanning speed	100 mm/s

**Table 2:** The properties of the silicon nitride used in the experiments.

Parameters	Values
Density	3.2–3.3 g/cm^3^
Hardness	90–92 HRA
Breaking strength	600–1200 MPA
Elasticity modulus	2.9–3.2 × 10^5^ MPa
Poisson ratio	0.25
Sublimation temperature	1900 °C
Specific heat	0.71 J/g·K
Heat conductivity	23–28 W/m·K

**Table 3:** The experimental parameters.

Experiment No.	Pulse Energy (μJ)	Scan Spacing Δd (μm)	Included Angle β (°)	Scan Number
1, 2, 3, 4,	10	5	0, 30, 60, 90	1
5, 6, 7, 8, 9	5, 10, 15, 20, 30	20	90	1
10, 11, 12, 13	10	2, 5, 15, 20	90	1
14, 15, 16, 17	10	5	90	1, 5, 15, 25

## Data Availability

All data in support of the findings of this paper are available within the article.
